# New drug therapies, dedicated specialists and desperately-ill patients—but who will fly the flag for service developments in lung cancer as NHS budgets come under pressure?

**DOI:** 10.3332/ecancer.2011.209

**Published:** 2011-02-22

**Authors:** S Pinn

The challenge facing lung cancer specialists in the UK is daunting. While good progress is being made in some areas of management, the level of improvement in others is less impressive. Indeed, only 51% of lung cancer patients currently receive any kind of active treatment. In some Trusts, it is less than 10%.

Investment in lung cancer is clearly disproportionate to the money spent on investigating its causes and that invested in potential therapeutic interventions. It is implicated in 22% of all cancer-related deaths in the UK, but attracts only 5% of total oncology research funding.

Meanwhile, lung cancer is the second most common cancer in the UK, with nearly 40,000 new cases diagnosed a year, according to the most recently available statistics.

New prescribing strategies based, for example, on the identification of genetic mutations in individual patients and differentiating between squamous and non-squamous histology, may help to make better use of new targeted therapies in the future—and even more cost-effective use of well-established cytotoxic chemotherapy regimens.

Whether or not the NHS will be able or willing to support such initiatives is another question altogether.

These were among the issues that emerged during *Oncology Outcomes—Taking Control of Tomorrow*, a meeting of oncologists and other health-care professionals with an interest in prescribing and service provision for lung cancer patients, held at the Royal College of Physicians in London on 5 November 2010.

Dame Gill Oliver (Immediate Past-Chair of the UK Lung Cancer Coalition) highlighted the following concerns:

- Poor communication, within and beyond the NHS
- Fear and guilt on the part of patients—especially among those whose lung cancer was known to be linked to tobacco smoking
- Varying levels of interest among health-care professionals
- Ineffective action by multi-disciplinary teams assigned to provide optimum and ongoing care
- Insufficient research into lung cancer and its causes
- Low level of patient expectations

‘We must pay far more attention to the ‘forgotten’ patients’, she insisted. ‘That means those who fall between the two extremes of being referred immediately for curative treatment and those for whom palliative care is the only realistic option’.

For the future, Dame Gill said that priority should be given to greater public awareness of the burden of lung cancer, more effective diagnosis, access to optimum treatments and top-quality specialist follow-up of patients. Meanwhile, lung cancer research should focus on screening patients to identify those most likely to benefit from specific therapeutic options, predictors of success, pulmonary rehabilitation, radiotherapy and better imaging techniques.

Particular attention should be paid in the future, she said, to the identification and measurement of outcomes rather than processes, and listening to what patients actually want. New therapeutic options, specialist training, as well as specific and effective diagnostic tests should be high on the agenda.

In order to address these questions, Dame Gill called for a ‘concerted and aspirational’ approach to delivering services—sharing best practice in order to raise the profile of lung cancer. ‘We need to be able to deliver lung cancer treatment to the level of the best that is currently available’, she concluded (Figure 1). ‘Improving access to diagnosis and early treatment will be the key to this goal’.

Dr Jesme Fox (Medical Director, Roy Castle Lung Cancer Foundation) drew attention to the fact that one of the main reasons for ambivalent attitudes towards lung cancer is that in countries such as the UK, where fewer people are now smoking, there is little sympathy for sufferers with a disease that is mainly caused by cigarette smoking.

Furthermore, she said, the very nature of lung cancer—either patients who are very ill or who have already died within a short time of being diagnosed—meant that are few patient advocates to advance the lung cancer cause. Dr Fox reported that her own organisation is the only national lung cancer charity registered in the UK—in sharp contrast to the prolific charitable provision enjoyed by other cancers.

Turning to the anticipated squeeze on NHS budgets, Dr Fox said there would be no escaping painful decisions on how best to make savings in order to fund service developments in lung cancer. There might include:

- reducing referrals to secondary care in those circumstances where GPs have better direct access to diagnostic testing
- reducing the level of ‘routine’ patient follow-ups
- exploring initiatives to reduce hospital admissions and length of stay

She outlined the RCLCF’s key campaigning priorities, including better research into early detection, efforts to reduce the stigma and negativity associated with lung cancer, access for all patients to lung cancer clinical nurse specialists, ensuring high-quality data collection through the National Lung Cancer Audit, speedier referral to a hospital-based specialist for investigation of suspicious symptoms, and greater equity throughout the UK in terms of early diagnosis, treatment and care.

Dr Fox concluded: ‘We live in challenging times. Lung cancer is a devastating disease, characterised by late diagnosis, few curative treatment options and poor survival rates. By engaging with patients, lung cancer specialists and other health-care professionals can make a more effective contribution towards reversing these trends’.

Professor Nick James (School of Cancer Sciences, University of Birmingham) reported that half of all drugs in clinical trials are for cancer indications. Rapid growth of cancer drug sales was a feature of the late 2000s, resulting in a global market worth $48 billion in 2008. Even biotech companies with no licensed products could be worth many billions of pounds, he said—in the expectation that they would find the key to the latest blockbuster.

The problem, however, was that new cancer drugs were usually only licensed (initially, at least) in end-stage disease—and that although they might improve survival, there was a high price to pay for any clinical benefit.

Despite the Government’s contention that NHS spending is ‘protected’, Professor James warned that in the UK, the National Institute of Health and Clinical Excellence (NICE) is likely to reduce its quality-per-life-year threshold substantially, thereby dashing any hope of adequate spending on new cancer lung cancer drugs in the future.

He pointed out that NICE has no budget for implementing its recommendations, and that during November 2010 doubts were raised about its role in terms of funding the prescribing of new drugs.

‘As it is, the UK currently spends far less on new targeted therapies than other European Union countries—and spending on cancer drugs continues to be driven almost entirely by the use of much older agents in adjuvant and neo-adjuvant settings’, he said.

Professor James added that although NHS spending on cancer drugs multiplied sixfold between 1998 and 2007, and that approvals for new cancer drugs are expected to continue, it was clear that future budget restrictions would soon put a brake on this rate of increase.

It might be possible to save costs by adhering to guideline-driven medicine, better screening and detection, and more effective disease profiling guided by risk-directed management. For the immediate future, the goal would be to improve cure and life prolongation rates in cancer. Nowhere was this need more urgent than in lung cancer, he concluded.

Professor Nicholas Thatcher (Christie Hospital NHS Trust, Manchester) outlined current thinking in terms of first-line therapy for in advanced non-small cell lung cancer (NSCLC). He said that a ‘therapeutic plateau’ had now been reached in terms of chemotherapy for advanced NSCLC—median survival platinum doublet chemotherapy achieving a median survival of 8–10 months in the late 2000s, compared to just 2–5 months in the mid-1970s.

He predicted that in the future, the pathologist would take centre stage in determining which patients would benefit most from specific therapeutic regimens for advanced NSCLC, highlighting the fact that outcomes—even with new targeted therapies such as bevacizumab and gefitinib—differ significantly when a distinction is made between squamous and non-squamous histologies.

Attention was now turning, said Professor Thatcher to first-line clinical trials with conventional therapy in combination with one or more of the new targeted therapies. For example, in studies such as SAiL (Safety of Avastin in Lung) [1] and ARIES (Avastin Registry: Investigation of Effectiveness and Safety) [2], a combination of doublet platinum-based chemotherapy in combination with bevacizumab had achieved consistently high median survival rates of 13.6–14.6 months.

He also cited recent data from IPASS (IRESSA Pan-ASia Study) [3,4], comparing gefitinib with platinum-based doublet chemotherapy in advanced NSCLC, to demonstrate that predictive markers such as activating epidermal growth factor receptor (EGFR) mutations not only determine whether or not a particular intervention has a beneficial impact on conventional endpoints such as progression-free survival (PFS), but importantly on parameters such as quality of life (QoL) and symptom improvement.

‘For patients with lung cancer’, said Professor Thatcher, ‘PFS *per se* is not necessarily relevant. It is not simply a matter of how long they live without progression, but how well they can expect to enjoy any extra months of life that result from a chosen intervention’.

In IPASS, he reported, significantly more patients with EGFR-positive mutations had sustained and clinically-relevant improvements in terms of QoL and symptom control with gefitinib than with doublet chemotherapy. For patients who were EGFR mutation-negative, the reverse was true.

Professor Thatcher went on to describe a relatively new chemotherapeutic agent, pemetrexed, as a ‘UK success story’ in an era when the UK was still being pilloried for its low lung cancer survival rates. ‘Who would have thought that a cytotoxic drug would have made such a difference in the treatment of advanced NSCLC’, he said.

Clinical data showed conclusively that in patients with non-squamous advanced NSCLC, pemetrexed in combination with cisplatin significantly improved survival without grade 3/4 toxicity compared to gemcitabine/cisplatin—again proving the value of differential histology.

Dr Marianne Nicolson (Aberdeen Royal Infirmary, Scotland, UK) argued the case for greater use of personalised therapeutic interventions and more discriminating patient selection, with the emphasis on maintenance and second-line treatment for patients with advanced NSCLC.

She reported that during the 2000s, clinical trials involving cytotoxic drugs such as gemcitabine, paclitaxel and docetaxel had all been studied extensively, but had not found favour as candidates for maintenance therapy in routine clinical practice—probably because although significantly longer PFS had been documented, there were only modest improvements in overall survival (OS) as well as increased adverse events and impaired QoL.

‘Are we any better off in the brave new world of targeted drugs?’ asked Dr Nicolson. ‘Maybe not’, she said. She reported the outcomes of a number of trials such as AVAIL (AVAstin in Lung) [5], FLEX (First-Line Erbitux in Lung Cancer) [6], ESCAPE (Evaluation of Sorafenib, Carboplatin And Paclitaxel Efficacy in NSCLC) and WJTOG 0203 [7] where agents such as bevacizumab, cetuximab, sorafenib and gefitinib, respectively, been had been added to conventional chemotherapy in the maintenance setting—all with little evidence of clinical benefit in terms of survival.

In SATURN (Sequential Tarceva in Unresectable NSCLC) [8], however, OS improved in patients receiving maintenance erlotinib who had not progressed on first-line chemotherapy—a benefit seen both in patients with wild-type (normal) and with mutated EGFR status (*p* = 0.0014 and *p* = 0.0019, respectively, versus placebo). In squamous patients who had not progressed, the survival benefit was also significant (11.3 months versus 8.3 months for placebo, *p* = 0.0116).

Finally, Dr Nicolson reported data to suggest that delaying treatment with a chemotherapeutic agent such as docetaxel not only achieves no significant clinical benefit in terms of OS, it also increases the risk of the patient not being fit enough for maintenance therapy [9].

Dr Nicolson concluded that:

- Most positive chemotherapy maintenance studies are in the early second-line setting, therefore it is important to start such therapy as early as possible.
- With targeted drugs, there is no worthwhile improvement for induction followed by maintenance (although erlotinib improves survival post-stable disease following induction chemotherapy.
- Parameters such as performance status, gender, best response, histology and molecular markers—including EGFR and vascular endothelial growth receptor—should all be taken into account when selecting patients for personalised therapy.

Dr Sanjay Popat (Royal Marsden Hospital, London) and Dr Riyaz Shah (Kent Oncology Centre, Maidstone and Tunbridge Wells NHS Trust) outlined recent developments that may well have a bearing on future prescribing and strategy for patients with lung cancer.

Dr Shah reported data from the IFCT-0501 trial showing that doublet chemotherapy (carboplatin + paclitaxel) provides better response rates in elderly patients (70–89 years), with more manageable toxicity than single agent chemotherapy (either vinorelbine or gemcitabine) [10]

Dr Shah then highlighted impressive clinical activity and rapid symptom relief with crizotinib, a novel inhibitor of another newly-identified molecular marker (EML4-ALK) in patients with NSCLC—especially in never smokers [11]

He also reported a positive impact of early palliative care on QoL, reducing the need for aggressive chemotherapeutic intervention at the end-of-life and improving survival in patients with stage IV NSCLC [12]

Finally, Dr Popat reported encouraging efficacy from the LUX-Lung 1 trial [13] with a new, potent and irreversible EGFR-inhibitor, afatinib, in patients with stage IIIb/IV adenocarcinoma of the lung—improved disease control rate (58% versus 19% compared to placebo) and prolonged PFS (from 3.3 to 1.1 months).

However, as Dr Shah made clear, the future for UK practice in terms of delivery of lung cancer care is likely to become increasingly complex. ‘EGFR and EML4-ALK are just two of a number of molecular markers now on the horizon’, he said. ‘Not only are new targeted therapies becoming available, but there are new chemotherapeutic agents and new indications for old chemotherapy regimens’.

## Panel

Highlights from interactive keypad voting at the Oncology Outcomes meeting:

- More than 73% of those who voted agreed that the media should be encouraged to highlight ‘innocent’ cases of lung cancer (e.g. women and non-smokers)) as a way of achieving more research and treatment resources.
- Nearly 70% argued that more campaigning is needed so that lung cancer gets a fairer share of the current cancer research funding pot.
- More than 70% said it would be reasonable to routinely request EGFR mutation testing in non-squamous patients.
- 50% recommended second-line treatment at progression.
- 56% believed that for the patient, the most important endpoint is survival, while 44% said it was freedom from symptomatic progression.
- 86% were of the view that people should be encouraged to insure against the need for co-payment with the NHS for access to cancer services.
- Two-thirds of responders said that it would not be possible to deliver the complexity of lung cancer care required in the future at every district general hospital.

Key points from the post-meeting debate, chaired by television and radio presenter Michael Buerk:

## Smoking and early diagnosis

- ‘It would be wrong for us to be more sympathetic towards women and non-smokers than we are towards smokers. For a charity such as ours, that would not be a responsible approach’. – Dr Jesme Fox (Roy Castle Lung Cancer Foundation)
- ‘Using the example of a 70-year-old, social class 4 or 5 male smoker is unlikely to create much sympathy for the lung cancer cause, but at the same time there is a certain amount of ‘dishonesty’ in trying to create a more attractive image of lung cancer’. – Dr Marianne Nicolson (Aberdeen Royal Infirmary)
- ‘I understand the anxieties—however, the implied dishonesty may be a pragmatic way forward. Lung cancer sufferers who have been smokers will benefit if there is greater sympathy for lung cancer sufferers in general’. – participant
- ‘Nevertheless, there needs to be a clear message that smoking causes lung cancer. Furthermore, by the time patients present with symptoms, they will already have stage IV disease. We need to use screening at an earlier stage to identify these cases before it is too late to intervene’. – Dr Riyaz Shah (Kent Oncology Centre)
- ‘We must focus on stopping young people from smoking in the first place. After the first 100 cigarettes, they are already hooked’. – Professor Nicholas Thatcher (Christie Hospital NHS Trust, Manchester)

## New drugs and equity of access to cancer services

- ‘While I accept that the NHS cannot be expected to pay for every new drug that is approved, there must be equity of access to what is deemed to be affordable. That is paramount’ – Dr Riyaz Shah (Kent Oncology Centre)
- ‘I am not convinced that the survival benefits of many of these new drugs justifies the expense of being able to prescribe them’. – Professor Nick James (University of Birmingham)
- ‘Funding for the testing of new drugs in the UK is a bit of as shambles. It’s an issue that is not even considered by some Cancer Networks’. – participant
- ‘Tailoring treatment for individual patients may be a way of freeing-up budgets to spend more on new drugs’. – participant
- ‘Perhaps patients should be encouraged to go the extra mile to get to a specialist centre—at least for the first consultation—rather than expect to get an expert opinion from their local DGH’. – Professor Nicholas Thatcher (Christie Hospital NHS Trust, Manchester)

## Clinical trials

- ‘The pharmaceutical industry is unwilling to undertake its pivotal trials in the UK—with the result that UK clinicians are not getting first-hand experience of whether or not these new drugs are of benefit’. Professor Nicholas Thatcher (Christie Hospital NHS Trust, Manchester)
- ‘There is no continuity from the pre-clinical development of new drugs through to phase III clinical trials’. – participant
- ‘New biomarkers are not being evaluated to the same standard as EGFR’. – Dr Sanjay Popat (Royal Marsden Hospital, London)

## Co-payment for new drugs

- ‘Reluctantly, I have to agree that co-payment may be a solution to the current dilemma. Most new drugs are not affordable within the current economic paradigm operating within the NHS’. – Professor Nick James (University of Birmingham)
- ‘An insurance policy for the price of a pack of cigarettes—that can’t be a bad deal’ – Dr Marianne Nicolson (Aberdeen Royal Infirmary)
- ‘It is the fundamental intention of the NHS to follow the co-payment line. In fact, it’s already begun. You get what you pay for in life. In a consumer-driven society, those who have access to cash will pay for the privilege of access to new drugs for lung cancer’. – Dr Riyaz Shah (Kent Oncology Centre)

## Figures and Tables

**Figure 1:**
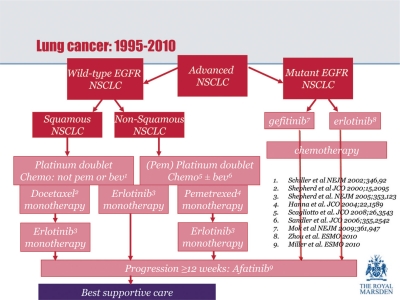
Lung cancer treatment—suggested pathway.
